# New Drugs, Appliances, and Things Medical

**Published:** 1889-10-12

**Authors:** 


### NEW DRUGS, APPLIANCES AND THINGS MEDICAL.

### "CHRISTIA" AND MODERN-DAY POULTICES.

In the museum of the British Medical Association's meeting at Leeds, the enterprising firm of Christy and Co. showed a large and comprehensive exhibit. Amongst other new things was a waterproof tissue for surgical dressings, called "Christia," which is intended to replace the old-fashioned oilskin and the rubber tissues of modern days at a much less cost. There is certainly room for such an article, for hitherto the high price has been a serious hinderance to the general adoption of waterproof materials. Their action in antiseptic surgery is twofold—protective against entry of germs from without, and preventive against escape of volatile antiseptic substances from within. But further important purposes can be served. By stopping the evaporation of wet dressings, any given part of the body may be kept warm and moist after the fashion of a poultice, which constitutes, broadly speaking, a local vapour bath. Should this kind of action not be needed, it is merely necessary to put some dry absorbent antiseptic material beneath the waterproofing in place of the wet application. Under a square, say, of "Christia," place a smaller piece of Gamgee tissue, and you make a perfect antiseptic dry dressing. Cover in the same way a bit of lint, wrung out of warm water, and you have a poultice after the modern method.

Nothing in the whole history of medicine as an applied science can be more remarkable than the modern developments of therapeutic appliances. One after another the old bugbears of our youth have disappeared for ever into the shades of oblivion. The nauseous potions of former days have to a great extent been replaced by pleasant or tasteless preparations, and that most discriminating of mortals, the budding schoolboy, is no longer justified in applying to his physic the terse and favourite expression "beastly." It is true that the uneducated, possibly from long generations of tolerance, still cling to their quassia and other abominations, but the physician of the world must be nowadays above all things a master of so-called "elegant pharmacy."

Over all the "bogies" of our youth one looms large and ghastly. It is the old familiar poultice, now, thank goodness! doomed to die the death of extinction before the advance of therapeutic methods. What visions does the memory recall! The scalded foot of the nursery, treated with bread poultices. At first they were applied one after another every hour punctually as clockwork, but soon, with the lessening of the pain, that attention waned, and at last half-a-dozen hours were allowed to go by without a change. On waking, that "cataplasm," as the old family doctor used to call it, had invariably shifted from its place and grown cold and stiff. And then there was that other horror, the "jacket-poultice," made inches thick and wrapped round the catarrhal chest. Could mother and nurse but reflect that in these cases breathing is already impeded by the lung affection, they would see that a heavy poultice must be a serious addition to the difficulty. Suppose in a weak child the movements of the abdomen to be further hampered by tight clothing, then it is no wonder should the infant fail altogether in the attempt to draw the breath of life into its unfortunate body. Anyone who has seen much practice among the poor will be able to testify to the truth of this picture of childhood fighting for breath sheathed in a cuirass of linseed meal.

Poultices badly made, were they? And, pray, can any reasonable being expect a professional poultice-maker to be attached to every household? Hardly, but modern methods go far to shelve the necessity. Abernethy is the man everyone thinks of in connection with poultices. He was said to "actually revel" in the idea of those interesting and, at that time, invaluable weapons of surgical treatment. His directions for scalding, mixing, stirring, spreading, folding, and applying his favourite "cataplasms" are numerous and exact. But extinction now threatens the whole army; linseed meal, bread, poppy-heads, camomile, potato, turnip, carrot, charcoal, and the fifty-and-one others that have been in vogue. Even the old familiar mustard is replaced by the prepared "leaf," which is not only cleanly, but can be kept at hand ready for use at a moment's notice. Poor people, no doubt, will still continue to use their mustard poultice (or "blister") just as they will cling to their linseed meal and other relics of a long-suffering past. And medical men in club, dispensary, and hospital practice will go on ordering poultices just as they will prescribe "beastly" medicines. Popular taste demands treatment that appeals to the senses, and a feeling of that kind must be respected in practice, or the physician, however skilful and conscientious he may be, will soon find himself on the wrong side of the hedge. But, as before hinted, the case is different in private practice, where one has to deal with educated people, who do not, as a rule, want to prescribe for themselves, and are readier to obey directions.

What then is to replace the poultice?

Why, simply a wet application, medicated or not, covered in by a waterproof tissue to prevent evaporation. The method of action is briefly as follows: the heat of the body causes the fluid to evaporate, but the "steam" or vapour is prevented from escaping by the outer rubber material, so that the part beneath the dressing is kept warm and moist just as by a poultice, only with the difference that the latter is liable to get hard and stiff and cold, and is difficult to keep closely applied to the body. But take our modern water dressing, a bit of lint dipped in warm water, place it over the desired spot, and cover with a rather larger piece of elastic tissue, bandage the whole securely on, and the thing is done. What could be quicker, neater, or simpler? Spongiopiline is on the same principle. It is a thick, spongy felt waterproofed on the outer surface, and can be medicated, if wished, before application. This material, however, like many other good things, is somewhat costly; the same objection that has hitherto applied to the various rubber and oiled silk tissues in use for surgical purposes.

And now, lest readers should think all the above to be too much like revolutionary and radical overstatement, let a little word be murmured on the other side. Poultices have done yeoman service in their time, and no doubt will do so still for many a long day. It is probable that for a certain class of cases, such as, for instance, old-standing ulcers, and things of that kind, the old-fashioned linseed-meal poultice will hardly be improved upon. When well made, that is to say, of proper consistency, smoothly mixed, thinly and evenly spread, nothing can equal the feeling of comfort and support derived by the patient from such an application. The poultice is likely to die as hard a death as the bleeding-lancet. Yet, for all that, rubber tissue will be found to give, with few exceptions, a cheaper and more manageable "local bath."

